# Patient autonomy versus family centric decision making in end-of-life care

**DOI:** 10.12669/pjms.41.13(PINS-NNOS).13504

**Published:** 2025-12

**Authors:** Raana Shahid, Manahil Irfan, Syed Shahzad Hussain Shah

**Affiliations:** 1 Dr. Raana Shahid, MBBS. Punjab Institute of Neurosciences, Lahore, Pakistan; 2 Dr. Manahil Irfan, MBBS. Agha Khan University Hospital Karachi, Pakistan; 3 Dr. Syed Shahzad Hussain Shah, FCPS. Punjab Institute of Neurosciences, Lahore, Pakistan

**Keywords:** Advance Care Planning, Decision Making, Palliative Care, Personal Autonomy

The global cancer burden is rising, with CNS tumors becoming a major cause of mortality. The Central Brain Tumor Registry (CBTRUS) reports a CNS tumor incidence of 24.83 per 100,000 annually, with 6.94 malignant and 17.88 non-malignant cases. Incidence is greater in females and non-Hispanic individuals.^1^ Glioblastoma is the most common malignant tumor, while meningioma leads among non-malignant types. From 2016-2020, CNS malignancies caused 86,030 deaths. The five-year survival rate is low, with glioblastoma having the worst prognosis. Survival is highest in children (0–14) but declines with age, especially after 40.^1^

Palliative care supports patients and families by addressing concerns, providing counseling, and ensuring compassionate care. It manages pain while also caters to emotional, psychological, and spiritual needs, providing comfort during challenging times. Sheridan et al. from University of California reported that early palliative care lowers healthcare costs in advanced cancer, with greater savings when started before the final week of life.^2^ A terminal illness impacts both patients and their families, disrupting routines and causing physical challenges like pain, fatigue, and dependency on assistance. This loss of autonomy may be emotionally taxing as the patient may struggle with both physical discomfort and the emotional weight of needing assistance. High treatment costs can lead to income loss and financial strain, with Walker-Pow et al. (2024) reporting severe financial insecurity in 23% of terminally ill patients.^3^ The psychological toll is severe, with patients experiencing depression, anxiety, and grief, and families facing caregiver burnout. A Swiss study found that requests for hastened death or MAID often arise from dignity and existential concerns such as loss of autonomy and feeling burdensome rather than physical symptoms.^4^

End-of-life (EOL) care focuses on comfort and pain management for terminal patients. While discussions allow patients to express care preferences, acknowledging medical limits and mortality remains challenging. A study done in 2008 in Boston indicates that both doctors and patients often feel hesitant and may avoid these discussions.^5^ Respecting the wishes of terminal cancer patients is vital. Advance care planning allows patients and families to outline priorities and preferences. In Western contexts, patients are often directly involved, whereas in many Asian cultures, families guide these decisions due to traditions and taboos around death. This can delay discussions, leaving key choices to doctors and relatives rather than the patient.^6^

Debate continues over who should guide end-of-life decisions. A 2022 Ugandan study showed that while patients value prognosis discussions, many prefer control over their timing and course, with cancer patients engaging at defined illness stages.^7^

Euthanasia is a contentious issue, banned in Islam but practiced globally, with ethical, legal, and cultural views varying on its legitimacy. The article discusses passive euthanasia and critiques the arguments made by the European Association of Palliative Care’s Ethics Task Force, which claims that “passive euthanasia” is a contradiction in terms. The paper argues that passive euthanasia—where life-prolonging treatments are withdrawn to hasten death in a patient’s best interest—can be ethically justified, especially when the patient’s quality of life is poor.^8^ A *New York Times* article highlights Derek Humphry’s euthanasia advocacy, citing his role in helping his terminally ill wife end her suffering. As a pioneer of the right-to-die movement, his book *Final Exit* significantly influenced the euthanasia debate.^9^

In Western contexts, end-of-life care emphasizes patient autonomy, while Asian traditions favor family-centered decisions rooted in respect for elders and cultural taboos around death. Physicians may also hesitate, fearing it signals loss of hope. Though this approach offers support, it can restrict patient voice and lead to aggressive treatments. With CNS tumors carrying high mortality, early palliative care can ease symptoms and emotional strain, yet social taboos, limited advanced care planning (ACP) awareness, and religious beliefs make such discussions difficult. Debates on euthanasia further underscore these ethical and cultural challenges.

Compassionate EOL care requires balancing patient wishes with family and cultural values. WHO should set global guidelines and integrate palliative care into Universal Health Coverage, while governments enforce ACP policies, expand training, and raise awareness. Physicians must use culturally sensitive communication, start conversations early, and ensure timely referrals. Ethical frameworks should be updated to reflect evolving values while safeguarding dignity and rights.

## Author`s Contribution:

**RS:** Concept and design of study and drafted the manuscript.

**MI:** Data acquisition and analysis and drafted the manuscript.

**SSHS:** Interpreted data, critically reviewed the manuscript and supervised the study.

All authors have approved the final version to be published and agreement to be accountable for all aspects of the work in ensuring that questions related to the accuracy or integrity of any part of the work are appropriately investigated and resolved.

## References

[1] Ostrom QT, Cioffi G, Gittleman H, Patil N, Waite K, Kruchko C (2019). CBTRUS statistical report:primary brain and other central nervous system tumors diagnosed in the United States in 2012–2016. Neuro Oncol.

[2] Sheridan PE, LeBrett WG, Triplett DP, Roeland EJ, Bruggeman AR, Yeung HN (2021). Cost savings associated with palliative care among older adults with advanced cancer. Am J Hosp Palliat Care.

[3] Walker-Pow R, Bruun A, Kupeli N, Bosco A, White N (2024). A systematic review on the impact of financial insecurity on the physical and psychological well-being for people living with terminal illness. Palliat Med.

[4] Seiler A, Amann M, Hertler C, Christ SM, Schettle M, Kaeppeli BM (2024). Effects of dignity therapy on psychological distress and wellbeing of palliative care patients and family caregivers –a randomized controlled study. BMC Palliat Care.

[5] Wright AA, Zhang B, Ray A, Mack JW, Trice E, Balboni T (2008). Associations between end-of-life discussions, patient mental health, medical care near death, and caregiver bereavement adjustment. JAMA.

[6] Kim H, Im HS, Lee KO, Min YJ, Jo JC, Choi Y (2021). Changes in decision-making process for life-sustaining treatment in patients with advanced cancer after the life-sustaining treatment decisions-making act. BMC Palliat Care.

[7] McEvoy P (2015). Euthanasia, ethics, and the Gordian knot:is the Hippocratic code obsolete?. Br J Gen Pract.

[8] Owusuaa C, van Lent LGG, van 't Spijker A, van der Rijt CCD, van der Heide A (2022). Discussing prognosis and the end of life with patients with advanced cancer or COPD:a qualitative study. PLoS One.

[9] Grimes W (2025). Derek Humphry, advocate for assisted suicide, dies at 93. N Y Times.

